# Annual Banquet and the Texas State Medical Association

**Published:** 1889-06

**Authors:** 


### ANNUAL BANQUET AND THE TEXAS STATE MEDICAL ASSOCIATION.

### LETTER FROM EX-VICE PRESIDENT O. EASTLAND.

*Editor Daniel's Texas Medical Journal:*

There are times when one's voice influences none, yet the time may be one when he should speak by way of alignment. Such being my feeling in taking the pen, I am sure of accomplishing one if I fail in the other.

The scenes of our recent meeting in the hospitable city of San Antonio are too fresh in our minds to need reference, and the question of annual banquets has been the topic of discussion for several years past. True, it has always been in the very best of feeling by those differing in opinion, just as all subjects should be dealt with in the organized profession of the State.

Coming at once to the question, we ask: Can the better interests of the Association be subserved with or without the annual banquet?

That over-indulgence in any way is harmful, is recognized more by our profession than any other; that there is over-indulgence in the "generous wine" at our banquets goes without question, hence it was thought best to prohibit wine at our banquets. Immediately this was objected to by local committees of arrangement, who said this trammeled them in their entertaining, and that other places where the Association had previously met were not thus limited in the matter of what they should place before their guests. Thus taking refuge, the question rested. But the adverse feeling, in the face of observation, has continued to grow. Then the propriety of leaving off the banquet entirely presented itself, and if I mistake not, the present sentiment of the Association is to omit the banquet in 1890, and in order to be sure, let's have a record of sentiment on the question now; that we begin in the very commencement of the new year, so ample time will be afforded to raise our index fingers for the guidance of our brethren in Ft. Worth, than whom no better exist. I think the fingers will point rather to Pike's Peak than the banquet room.

